# Mothers Matter: Using Regression Tree Algorithms to Predict Adolescents’ Sharing of Drunk References on Social Media

**DOI:** 10.3390/ijerph182111338

**Published:** 2021-10-28

**Authors:** Sebastian Kurten, David Winant, Kathleen Beullens

**Affiliations:** 1 Faculty of Social Sciences, Leuven School for Mass Communication Research, KU Leuven, Parkstraat 45, 3000 Leuven, Belgium; kathleen.beullens@kuleuven.be; 2 Department of Electrical Engineering, Dynamical Systems, Signal Processing and Data Analytics (STADIUS), KU Leuven, Kasteelpark Arenberg 10, 3001 Leuven, Belgium; david.winant@esat.kuleuven.be

**Keywords:** machine learning, regression tree, CART, extreme gradient boosting, social media, adolescents

## Abstract

Exposure to online drinking on social media is associated with real-life alcohol consumption. Building on the Theory of planned behavior, the current study substantially adds to this line of research by identifying the predictors of sharing drunk references on social media. Based on a cross-sectional survey among 1639 adolescents with a mean age of 15 (59% female), this study compares and discusses multiple regression tree algorithms predicting the sharing of drunk references. More specifically, this paper compares the accuracy of classification and regression tree, bagging, random forest and extreme gradient boosting algorithms. The analysis indicates that four concepts are central to predicting adolescents’ sharing of drunk references: (1) exposure to them on social media; (2) the perceived injunctive norms of the mother towards alcohol consumption; (3) the perceived descriptive norms of best friends towards alcohol consumption; and (4) willingness to drink alcohol. The most accurate results were obtained using extreme gradient boosting. This study provides theoretical, practical, and methodological conclusions. It shows that maternal norms toward alcohol consumption are a central predictor for sharing drunk references. Therefore, future media literacy interventions should take an ecological perspective. In addition, this analysis indicates that regression trees are an advantageous method in youth research, combining accurate predictions with straightforward interpretations.

## 1. Introduction

In the past years, a substantial number of studies documented the association between social media use and alcohol consumption [1,2,3,4]. These studies show that adolescents’ interaction with media content depicting alcohol and drunkenness is a meaningful marker for offline drinking, which poses a severe threat to health during adolescence and young adulthood [2,5,6]. The portrayal of online drinking practices is a popular and well-orchestrated activity for young individuals [7,8,9]. Recent research has mainly looked at the content of these messages and their effects [2,10,11,12]. We have less insight, however, in the factors determining the sharing of these messages.

Few studies have examined the predictors of sharing alcohol related messages on social media. For instance, Litt et al. [10] examined young adults’ willingness to consume alcohol as a predictor of the sharing of alcohol-related Twitter messages. Geusens and Beullens [13] found sharing of alcohol-related messages to be associated with adolescents’ perceptions of how often their friends shared similar messages and personal alcohol drinking behavior.

In spite of the insights these studies provided, research lacks a systematic and thorough overview of the factors associated with adolescents’ sharing behaviors. Previous literature mainly looked at single factors associated with sharing of drunk references, but a systematic investigation of competing factors within a sound theoretical framework is currently missing. Consequently, building on Ajzen’s [14] theory of planned behavior (TPB), the first goal of the current study is to systematically examine predictors of sharing drunk references and compare their importance. Identifying central predictors is crucial in order to target future prevention efforts because social media interventions can focus more specifically on reducing their negative influence. The second goal of the current study is to improve the methodological development in the field by comparing the accuracy of different regression tree algorithms. The method is gaining momentum in survey research [15], since it can combine the predictive power of supervised machine learning (ML) with an easy to interpret underlying prediction model.

## 2. Theory of Planned Behavior

The use of alcohol and social media are big parts of adolescents’ lives. Recent surveys indicate that one out of three adolescents has engaged in binge drinking [16], and 95% use social media on a daily basis [17]. Sharing drunk references is a combination of both behaviors. Qualitative research revealed that the online portrayal of drunk references is a well-orchestrated activity among adolescents, aimed at creating an online identity that resonates with their peers. They spend a lot of time planning and creating pictures of themselves drinking in order to enhance popularity and belonging [9]. Therefore, the online sharing of drinking behavior is a planned behavior that incorporates intentional and social reaction processes.

The TPB [14] postulates that norms, attitudes, and perceived behavior control indirectly influence the likelihood to perform a specific behavior via behavioral intention. Norms reflect social influence on behavior. Injunctive norms refer to the perceived extent to which others approve of certain behavior, while descriptive norms are defined as an individual’s perception of how frequently others engage in a certain behavior [14]. They have been found to be linked to overall risk behavior [18], hazardous forms of drinking [1,19], and the sharing of alcohol references [20,21]. While numerous studies have mainly focused on the influence of peers and best friends [12,22], recent research has indicated that parental injunctive norms towards alcohol are also linked to the sharing of drunk references [20]. 

Attitudes represent an evaluation of specific behavioral outcomes, e.g., the attitude towards alcohol indicates whether an individual considers drinking a desirable behavior. Building on the TPB framework, past research has indicated that attitudes predict the intention to consume alcohol in the future [23]. However, slightly deviating from the original TPB framework [14], Gannon-Loew et al. [23] showed that attitudes toward the consumption of alcohol were also associated with the sharing of alcohol references on Facebook. In line with these findings, and because alcohol-related attitudes might be more salient compared to attitudes toward the sharing of alcohol-related messages, the current study will examine whether adolescents’ attitudes toward the use of alcohol predict the sharing of drunk references. 

The TPB identifies behavioral control as a key mechanism in predicting behavior. It refers to one’s belief about the ease or difficulties in actually engaging in that behavior. A related form of behavioral control, especially in youth research, is sensibility to peer pressure. It can be seen as the degree to which adolescents adjust their behavior in line with perceived pressure from their peers. For instance, Teunissen et al. [24] found that sensibility to peer pressure moderated the effect on alcohol consumption from norms. Furthermore, Geusens et al. [25] concluded that it might have a moderating effect on sharing alcohol references. The current study also investigates related behaviors like social comparison orientation [26] and sensation seeking, because both forms of behavioral control are strongly linked to media use [27,28]. These concepts related to behavioral control do not represent the specific form of behavioral control that TPB postulates. Nevertheless, they have been identified by previous research to be influential in the initialization of risk behavior [24,25,28]. Sensation seeking is an especially influential predictor for media-related risk behavior, because adolescents showing a high level of sensation seeking select more risky media content [28], and might thus also share more risky content. 

Behavioral intention is the central mediator and antecedent of behavioral outcomes in the TPB [14]. However, beyond intention, other related indicators have also been found to predict behaviour. Specifically, willingness to drink alcohol has been identified as a predictor for the consumption of alcohol [29,30] and for the use of Twitter while drunk [10]. These findings provide a strong mandate to include the willingness to drink as a predictor for sharing drunk references in the current study. 

Prior literature used parts of the TPB to analyse the sharing of alcohol references [20,21,31]. For instance, Geusens and Beullens [31] predicted the sharing of alcohol references by peer feedback, while another study looked at the influence of parental norms on sharing alcohol references [20]. The current study adds to this line of research by including a wider range of behavioural predictors of the sharing of drunk references. It is essential to know which concepts are the most influential to tailor future prevention efforts effectively. This study will address this lacuna in the literature by identifying the most important predictors for sharing alcohol references. To do so, it will deploy advanced regression tree algorithms in an innovative way.

## 3. Regression Tree Algorithms

Regression trees are binary structured classifiers that repeatedly split a dataset into subsets [32]. They combine the predictive power of supervised machine learning (ML) with the interpretability of classic multivariate techniques, making them an ideal choice for ML applications. Breiman et al. [32] originally introduced classification and regression trees (CARTs) to predict binary or continuous outcomes. Building on that approach, Tin Kam proposed [33] the random forest algorithm. It generates several regression trees and uses the mean of the trees to predict an outcome. This procedure can be referred to as ensemble learning. Another recent increase in the popularity of regression tree algorithms can be attributed to the development of the Extreme Gradient Boosting (XGB) algorithm [34]. XG boosted trees were often used within the winning models of ML challenges initiated by websites like Kaggle, which is an important indicator for their potential. XG boosting refers the practice of weighting the predictions of multiple trees differently in order to increase precision, and is among the state of the art regression tree algorithms.

Regression trees allow for distinguished feature selection, making them the method of choice for the present study. By using the concept of information gain or entropy reduction from information theory [35], regression trees offer a way to select the most meaningful predictors in a model. We will use the approach to select the most influential norms and other predictors derived from the TPB.

This method is different from other studies designed to test the TPB to explain alcohol-related risk behaviour [36,37,38]. These studies usually deploy mediation or moderation analysis that focuses on theoretical inference to confirm or falsify hypotheses derived from the TPB. The current study, however, aims at predicting which adolescents are vulnerable to risk behavior by using regression trees. According to Bzdok et al. [39], machine learning methods are superior to classical statistical techniques like mediation analysis when aiming for most accurate predictions.

## 4. Materials and Methods

### 4.1. Study and Sample

The dataset is part of the first wave of the Belgian adolescents media investigation study (BAMI) among a sample of 1645 adolescents. Despite the legal drinking age of 16 in Belgium, a substantial number of adolescents engage in alcohol consumption before being legally allowed to do so [16]. To gather participants for the study, 41 school principals received emails explaining the study’s aims, practicalities, and ethical guidelines. Of the 41 schools, 18 secondary schools agreed to participate. Research assistants visited the schools and gave instructions on ethical guidelines to all participants (i.e., voluntary participation, anonymity, confidentiality). In order to not prime adolescents on the relationship between media and alcohol, we presented the study as an investigation on going out and leisure related activities. Upon granting active consent, high school students filled out a standardized paper-and-pencil questionnaire. This questionnaire was pretested among a small sample of adolescents to improve clarity and comprehension. The institutional review board of the authors’ university approved this study. The dataset of the first wave of the BAMI study, which was used for the current work, is available via the open science framework (https://osf.io/hxsv6/ accessed on 26 September 2021).

The amount of missing data in the overall dataset was rather low. The proportion of missings for sharing drunk references ranged from 1.7% (Instagram Chat) to 2.6% (Snapchat Snaps). We used the mice package [40] to develop a multiple imputation model to predict the missing values. Density plots were used to access whether the distribution of observed values fit the distribution of imputed ones. The imputed values were used for further analysis.

### 4.2. Measures

#### 4.2.1. Sharing of and Exposure to Drunk References on Social Media

In line with Geusens and Beullens [31], the sharing of and the exposure to drunk references was measured using seven point Likert scales with eleven items. Participants were asked: “How often do you share photos or videos where you or someone else is drunk or write a message while you are drunk through the following channels?” and “How often do you see photos or videos where someone is drunk or a message written by someone who is drunk through the following channels?” The Items were “Facebook timeline”, “Facebook stories”, “Facebook messenger”, “Instagram posts”, “Instagram stories”, “Instagram chat”, “Snapchat Snaps”, “Snapchat stories”, “Snapchat chat”, “WhatsApp groups”, “WhatsApp private messages”. Answering categories were “I do not use it (=77)” “Never (=0)”, “Less than once per month (=1)”, “Several times per month (=2)”, “about once per week (=3)”, “Several times per week (=4)”, “Everyday (=5)”, and “Several times per day (=6)”. In order to keep the results representative for the whole sample of adolescents and not only social media users, we treated respondents who did not use a certain platform as if they did not share drunk references on it. Finally, averaged sum scores were created to respect the additive nature of the items.

#### 4.2.2. Injunctive Norms towards Alcohol Consumption

There were four scales used to measure injunctive norms towards alcohol consumption among four different socialisation agents. These are the mother, the father, peers, and best friends. The four different scales had a similar wording and they consisted of respectively six items: “How would your father/mother/peers/best friends react if she/he/they knew that…” “You drank alcohol?”, “you drink alcohol every day?”, “you drink alcohol every weekend?”, “you were drunk?”, “you were so drunk that you fainted?”, and “you have drunk at least four glasses (girls)/five glasses of alcohol (boys) in two hours?”. Answer possibilities were ranged on a seven point Likert scale covering “strongly disapproving”, “moderately disapproving”, “slightly disapproving”, “neutral”, “slightly approving”, “moderately approving”, “strongly approving”. The scale provided adequate reliability (α [41] = 0.78, ω_1_ [42] = 0.84, Average Variance Extracted (AVE) = 0.54). There are two low loadings (0.35 & 0.32) for the items regarding drinking every day and fainting after drinking. These questions represent rather extreme circumstances, which may be a reason for low loadings. Standardized regression factor scores (proportional variance = 41.7%) will represent the scale.

#### 4.2.3. Descriptive Norms towards Alcohol Consumption

We also addressed descriptive norms of peers and best friends in separate scales, asking respondents how many of their friends and peers “drunk a full glass of alcohol”, “drink now and then alcohol”, “drink sometimes more than four glasses (girls)/five glasses (boys) of alcohol per occasion”, “drink often more than four glasses (girls)/five glasses (boys) per occasion”, “are sometimes drunk”, and “are often drunk”. The answer possibilities were “none”, “the minority”, “half of them”, “the majority”, and “almost everyone”. Reliability was high for descriptive norms of peers (α = 0.95, ω_1_ = 0.95, AVE = 0.72) and best friends (α = 0.93, ω_1_ = 0.93, AVE = 0.67). Here, we also computed standardized regression scores (proportional variance for peers = 67% and best friends = 72.3%).

#### 4.2.4. Willingness to Consume Alcohol

The scale measuring the willingness to consume alcohol was adapted from Gerrard et al. [43]. Participants were asked: “Imagine that you are with a group of friends and that alcohol is available. How willing would you be to do the following things?” The wording of the six items was: “Taste a sip when a friend offers you a glass of alcohol”, “Say “No thanks” when a friend offers you a glass of alcohol”, “drink a glass of alcohol with your friends”, “drink several glasses of alcohol with your friends”, “Keep drinking alcohol when you already feel a little drunk and your friends offer you another glass”, and “getting drunk with your friends”. The answers were assessed on a five point Likert scale. The wording of the answers was: “not willing at all”, “not really willing”, “perhaps willing”, “slightly willing”, “very willing”. The scale provided acceptable reliability (α = 0.90, ω_1_ = 0.91, AVE = 0.67). The loading for the second item was rather low (0.31). This may stem from the fact that it is reverse coded. The standardized regression factor scores (proportional variance = 64%) were used for further analysis.

#### 4.2.5. Social Media Use, Further Norms, Attitudes, Peer Influence and Sensation Seeking

Social media use was measured in four one-dimensional questions asking respondents how often they use Facebook, Instagram, Snapchat or WhatsApp. Answer possibilities ranged from “never” to “several times per day”. Each of the indicators were used for analysis.

Several scales were used to describe peer influence. The sensitivity to peer pressure was measured similar to the manner proposed by Santor et al. [44]. They have originally formulated ten items to measure peer pressure related to alcohol consumption, drug use, and sexual behavior. Of these ten items, we removed two items aiming at sexual behavior and drug use. Their wording can be found in the original publication [44]. Standardized regression factors scores were computed for the eight items.

The Brief Fear of Negative Evaluation Scale was used to measure fear of negative evaluation [45]. The scale’s twelve items were used to compute standardized regression scores. 

Social comparison orientation was measured in line with Buunk and Gibbons [46]. They proposed eleven items that were measured on a five point Likert scale. Their regression factor scores were used for further analysis.

Respondents’ sensation seeking behavior was assessed through the Brief Sensation Seeking Scale as proposed by Hoyle et al. [47]. They used eight items on a five point Likert scale. We summed up the scale by using regression factor scores.

Adolescents’ attitude towards alcohol was measured in line with Geusens and Beullens [3] on a semantic differential scale consisting of seven items. Respondents had to rate on a seven point scale whether “drinking alcohol is…” “abnormal or normal”, “harmful or unharmful”, “nice or dull”, “bad or good”, “cool or uncool”, “alternative or mainstream”, and whether it “makes things easy or makes things difficult”. For further analysis, regression scores of that scale were used.

Table 1 shows that most of the latent factors provided acceptable reliability except for sensation seeking, sensitivity to peer pressure, and social comparison orientation. Their indicators extracted on average less than 30% of variance. Standardized regression factor scores were computed for further analysis.

### 4.3. Data Analysis

The outcome variable of interest is the sharing of drunk references. All measurements described in the measurement section were used as input variables. First, binary recursive partitioning was used to construct a regression tree, meaning that the predictions are split into binary partitions going from the biggest partition to the smallest or, in terms of regression trees, from the root to the leaves. The algorithms of the tree package [49] and the rpart package [50] in R were used. Second, the tree was pruned with R^2^ as a pruning criterion. Pruning describes the procedure of determining an ideal number of partitions to balance explanatory value and complexity. Additional nodes were removed when they did not substantially increase R^2^. Third, we used the random forest algorithm [51] that samples with replacement different training sets from the training set. Using these sets to train a tree on each of them is referred to as bootstrapping. We aggregated these predictions in a procedure called bagging. Then we applied a full random forest, which is similar to bagging, but a subset of the variables of the training set is used for each tree. To extend the accuracy of predictions, we used the eXtreme Gradient Boosting algorithm (XGB) by Chen and Guestrin [34]. XGB weighs the samples and assigns higher weights to cases that are predicted suboptimally in order to improve the predictions (boosting). Gradient boosting using gradient descent optimizes this procedure further. It was implemented in the H_2_O ML platform [52], which also comes with an R interface [53]. All of the presented approaches were be applied to compare their prediction accuracy.

Supervised ML requires splitting the dataset into a training set (n = 819) and a test set (n = 820). The training sets served to optimize the parameters of our model, and the test set was then used to evaluate the results. To access the quality of the different models, we compared R^2^ within the training set and the MSE of predictions on the test set. After applying the analysis to the full dataset, the importance of the different variables was evaluated. A criterion for relative importance was the relative information gain.

It was of further interest to assess whether a more parsimonious model, only containing the most important variables, was sufficient to explain the regarded outcome variables. To do so, the analysis was rerun on a dataset containing only the variables that were part of the pruned tree. They were chosen based on R^2^ as it was used for pruning the tree, meaning that the analysis was rerun on a dataset only containing the factors “exposure to drunk references”, “willingness to drink alcohol”, “injunctive norms of the mother”, and “descriptive norms of best friends”. The results of both analyses were compared to check whether a subset of variables could predict the response variable with similar accuracy.

## 5. Results

Of the 1645 originally sampled adolescents, six were removed because they either did not want to or could not report on the injunctive norms of their parents. Table 2 shows that the remaining 1639 participants were on average 15 years old (SD = 2.38). There were a handful of outliers aged, 12, 19, and 20, which were kept in the sample. The sample contained 59% females. On average, adolescents indicated that they share drunk references never or less than once per month (0 corresponds to “never”, 1 to “less than once per month” and 2 to “several times per month”) (${\bar{x}}_{\mathrm{shr}}$ = 0.22, SD = 0.44), although they are exposed more often (${\bar{x}}_{\exp}$ = 0.76, SD = 0.78). The most frequent destinations for sharing drunk references are SnapChat snaps (${\bar{x}}_{\mathrm{shrSCsnp}}$ = 0.59, SD = 0.95) and SnapChat stories (${\bar{x}}_{\mathrm{shrSCstr}}$ = 0.36, SD = 0.81). The least popular destinations are Facebook’s stories (${\bar{x}}_{\mathrm{shrFBstr}}$ = 0.05, SD = 0.41) and timeline (${\bar{x}}_{\mathrm{shrFBtl}}$ = 0.06, SD = 0.38). WhatsApp’s group chat and private chat are also shunned outlets (${\bar{x}}_{\mathrm{shrWAgrp}}$ = 0.07, SD = 0.40 and ${\bar{x}}_{\mathrm{shrWAprv}}$ = 0.06, SD = 0.38). The exposure is highest on Snapchat stories (${\bar{x}}_{\mathrm{expSCstr}}$ = 1.48, SD = 1.41) and Snaps (${\bar{x}}_{\mathrm{expSCsnp}}$ = 1.33, SD = 1.38). WhatsApp’s private and group chat have low exposure on average (${\bar{x}}_{\mathrm{expWApriv}}$ = 0.29, SD = 0.74 and ${\bar{x}}_{\mathrm{expWAgrp}}$ = 0.23, SD = 0.78). Adolescents who did not use a specific platform or function were treated in the analysis as if they did not share or encounter any references.

**Figure 1:**
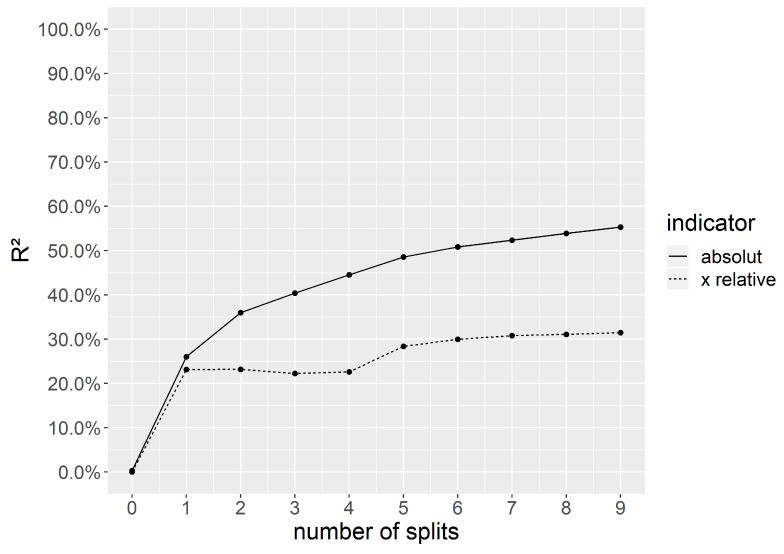
Determination of splits.

**Figure 2:**
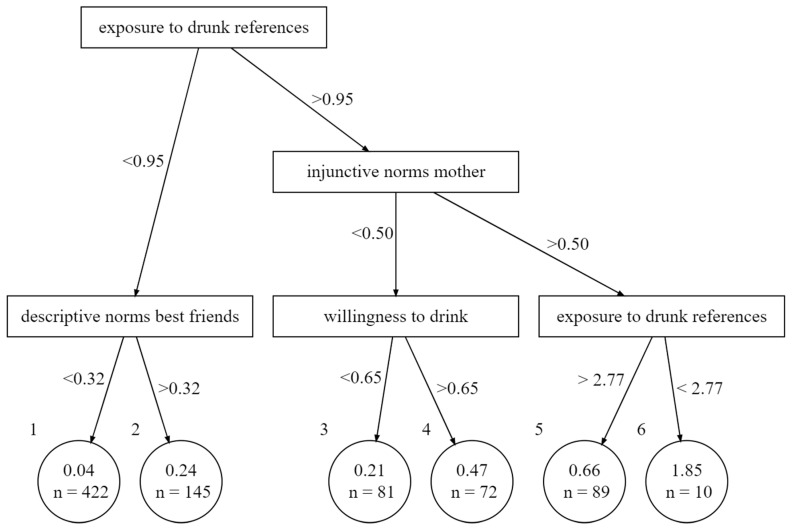
Pruned Tree.

Figure 1 shows that a pruned tree with five splits and six leafs provides a reasonable balance between complexity and interpretability. R^2^ does not increase substantially anymore after the fifth split and a further increase in splits would only increase complexity without gaining information. The model is able to explain 51.7% of the variance within the training dataset. The pruned tree is displayed in Figure 2. The node with the highest purity is the root representing the variable of exposure to drunk references on social media. It divides the training set into a group of 567 adolescents (nleaf1 + nleaf2) that are exposed to drunk references about less than 0.24 SDs above the mean, meaning that they are exposed to a drunk reference about once a month. The other group of 252 respondents is exposed more frequently than the split value. The 567 respondents can be further split according to the perceived descriptive norms of their best friends. If they score lower than 0.32 SDs above the mean, they belong to leaf 1, which represents the majority of 422 respondents who almost never share any drunk references. Leaf 2 represents 145 adolescents who score 0.24 on the averaged sum score of sharing, which is slightly higher than the variable’s mean (${\bar{x}}_{\mathrm{sharing}}$ = 0.21).

On the right side of the tree (Figure 2), mothers’ injunctive norms come into play. A high score indicates that someone perceives his or her mother to approve alcohol consumption among her children. If that value is above 0.5 SDs of the mean, the predicted frequency of sharing drunk references increases. If the exposure to drunk references is additionally 2.58 SDs higher than the mean, adolescents are predicted to share drunk references a little less than several times per month. Leaf six represents the ten adolescents that belong to the group of frequent sharers. If the mother has a higher disapproval, the willingness to drink is used again to classify respondents. Leaf 4 represents 47 respondents who have a higher willingness to drink than 0.65 SDs above the mean. Leaf 3 represents the 81 respondents for which that is not the case.

The pruned tree was used to predict the frequency of sharing drunk references in the test dataset. The mean square error (MSE) for these predictions is 0.170. The root of that value is the RMSE, which shows that predictions deviate on average 0.412 points on the scale of sharing drunk references from the test dataset. That is roughly equal to one SD (0.435) of the target variable. The RMSE is used to judge the accuracy of predictions because it is interpretable with regard to the outcome variable. Bagging was able to improve these predictions. The bagged trees reduced the MSE on the training dataset to about 0.09. The MSE on the test dataset dropped to 0.156. The application of the random forest algorithm did not improve the accuracy any further, whereas the application of XGB increased the prediction accuracy substantially. R^2^ in the test set decreased to 84.1% and the MSE dropped to 0.077, meaning that the model was able to predict the sharing of drunk references with an expected error rate of 0.637 SDs.

**Figure 3:**
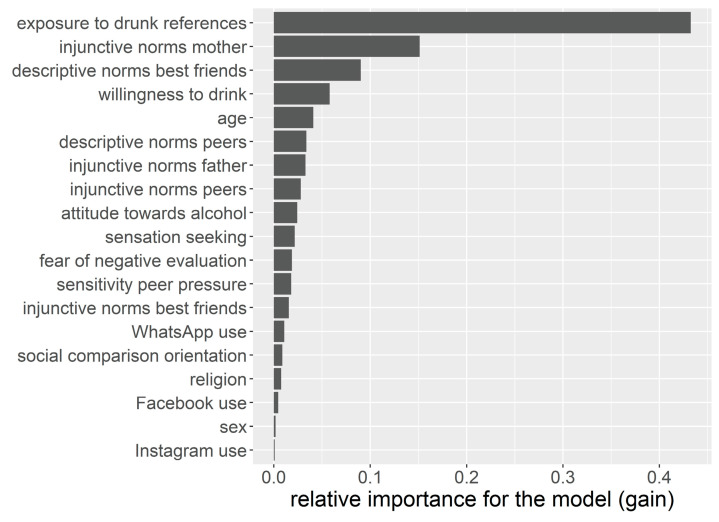
Relative Feature Importance.

Figure 3 shows the information gain of the different features for the XGB. It can be interpreted as the relative contribution of each variable for the models or, in other words, as the decrease in entropy after the dataset is split on a variable. By comparing relative information gain, we can examine the most important variables for predicting the sharing of drunk references. It shows that exposure to drunk references is the central prediction variable, followed by the perceived injunctive norms of the mother, the perceived descriptive norms of best friends, and willingness to drink alcohol. The use of different platforms, sex or religion did not contribute substantially to prediction accuracy.

To validate the predictive power of the most important variables, we reran the analysis with the four most important features. Table 3 shows a comparison of both analyses. Although, the smaller dataset can explain less variance within the training set, the accuracy is about the same for both sets. Again, the best results were obtained by using XGB. The model was able to explain 69.5% of the variance within the training dataset and the predictions yielded an expected error rate of 0.567 SDs for the sharing of drunk references in the test dataset, which is the most accurate result obtained in the Analysis.

## 6. Discussion

This work contributes to the literature in two important ways. First, it builds on theory of planned behavior (TPB) and past literature to predict adolescents’ sharing of drunk references on social media. It does so by using the relative feature importance defined as the decrease in entropy that the regression tree models cause in the dataset. This approach showed that the most important predictors of adolescents’ sharing of drunk references were exposure to drunk references, the perceived injunctive norms of the mother, perceived descriptive norms of best friends, and willingness to drink alcohol. The results provide implications for theory development as well as practical prevention efforts.

Second, by comparing several tree algorithms the study demonstrates the feasibility of using innovative machine learning (ML) approaches in youth research that combine the predictive power of computational ML approaches with the interpretability of multivariate statistics. The results have profound methodological implications for future research in the field of youth research.

### 6.1. Theoretical Implications

This study showed that maternal injunctive norms towards alcohol consumption, the perceived descriptive norms of best friends towards alcohol consumption, and the willingness to drink alcohol were the most influential concepts in predicting adolescents’ sharing of drunk references on social media.

Although past research has shown that the accessibility of norms is a central mechanism that translates them into action [54], the present study is among the first to indicate that perceived maternal norms are especially relevant when predicting the sharing of drunk references online. Stockdale and Coyne [55] argued that the decrease of parental control is a cause of increased media use during adolescence. Jackson et al. [2] concluded that parental control might also reduce the amount of adolescents’ alcohol exposure on social media. However, there might be differences between maternal and paternal influences. Elam et al. [56], for instance, found that the presence of a maternal alcohol disorder predicted adolescent alcohol use, while that was not the case for fathers. The results of the present study point in a similar direction by showing that maternal norms are also essential for predicting adolescents’ sharing of drunk references online. One potential explanation could be that mothers usually spend more time with their children than fathers and might therefore have a greater socialization influence [57]. Consequently, it seems plausible that maternal norms regarding alcohol use are more salient for adolescents and might, therefore, influence the sharing of drunk references.

The results also pointed towards the relevance of best friends’ norms. Extending the findings of previous studies on the influence of norms on adolescent risk behavior [18,54,58], the current work suggests that the salience of best friends’ norms is especially relevant in the context of social media-related risk behavior, that is, because it is interwoven with exposure to best friends’ risk behavior online. Adolescents share risk behavior online to send each other signals and connect with their friends [7], leading to an overstatement of its actual frequency that further amplifies its negative effect [59]. Consequently, the exposure to drunk references on social media and an increase in perceived descriptive norms co-occur with each other.

The current study was built on the TPB framework, but also deviated from it to a certain extent and was not designed to test TPB. In particular, the study used norms and behavioral intentions that referred to alcohol use instead of norms and intentions related to the sharing of drunk references online. In addition, the willingness to drink alcohol has been found to substantially predict the sharing of drunk references on social media. 

The study showed that alcohol related norms and willingness to drink are relevant considerations when explaining the sharing of drunk messages. In particular, the analysis showed that the salience of norms can differ. In a review of the social norms literature, Dempsey et al. [60] suggested that there is more research needed comparing the influence of injunctive and descriptive norms. The current analysis indicates that still further diversification might be necessary because the relationship between norms and risk behavior might differ among socialisation agents. Consequently, we propose that future research applying social norms within the TPB framework should account for this by comparing different norms to examine which norms are most relevant for a given behavioral outcome. This could be done by using norm salience as a moderator or comparing norms from different socialisation agents, and could generate valuable insights on the predictors of different forms of adolescent risk behavior.

Overall, the results of this work suggest that the social context of the adolescent in the form of best friends and mother is important when predicting sharing of drunk references on social media. These appear to be more important than behavioral dispositions like sensation seeking or the use of a specific social media platform. The present study suggests that investigating influences of different socialisation agents when reducing the sharing of drunk references can be a beneficial approach for future preventions.

### 6.2. Methodological Implications

The second aim of the article was to compare the precision of different regression tree algorithms. The analysis showed that advanced regression tree algorithms are beneficial in the analysis of survey data, especially when the researcher wants to identify the most important predictors for a given behavior. By allowing complex interactions between variables, they are superior to classical multivariate statistics in predicting actual behavior [39]. Consequently, their popularity has increased in survey research [15]. However, they are still rarely applied in youth research. We recommend future research to continue exploring the possibilities of regression tree algorithms, especially when scholars want to predict actual behavioral outcomes. Regression trees are an example of a method that balances predictive power with theoretical interpretability.

The study is among the first to compare regression tree algorithms for the analysis of survey data. The application of regression trees provided easily comprehensible results paired with high accuracy. The comparison to more advanced estimation algorithms showed that the Extreme Gradient Boosting (XGB) algorithm provided the most accurate predictions for sharing of drunk references. Previous comparisons of regression tree algorithms showed, for example, that XGB is among the most accurate algorithms in predicting survey non-response [15]. It does so by weighing suboptimally predicted cases higher, yielding two benefits. First, XG boosting is able to predict the behavioral outcome of individuals with a rare characteristic of variables, or outliers, better. Second, it reflects complex associations between variables, leading to higher ecological validity than traditional linear procedures.

### 6.3. Practical Implications

The findings of this study might help intervention efforts in identifying possible strategies for reducing risky social media use of adolescents. The results point towards two strategies.

First, in line with prior research on the significance of parental relationships in alcohol prevention [2,61], the results suggests that the influence of the mother can be used for reducing media-related risk behavior. Prevention programs could aim at making parents aware of the detrimental effects of sharing drunk references on social media. Adolescents might reduce their own sharing behavior if the mother (and the father) express a strong disapproval and educate about the negative consequences of sharing drunk references online.

Second, the application of media literacy interventions might be an effective strategy in reducing the likelihood of adolescents to share drunk references. Given that exposure to this content is a strong predictor for its sharing, it seems likely that media literacy interventions could reduce that negative effect. Due to promising results of media literacy interventions in reducing alcohol use of adolescents [62,63], we recommend testing them with the aim of reducing the sharing of drunk references as well. 

We encourage future prevention programs to combine both strategies, meaning that media literacy programs should also address the family system making use of an ecological perspective [64]. It is essential that the mother, and ideally both parents, participate in the education of their children about the negative consequences of sharing drunk references online.

### 6.4. Limitations

This study is subject to four major limitations. First, regression tree algorithms are rather sensitive towards small changes in the dataset. Given that, it may be possible that the algorithm produces a different tree for a slightly changed dataset. Consequently, more research is needed to validate the conclusions. Second, the study relied on self-reports. Despite the results of Flisher et al. [65], indicating that self-reports on adolescents’ risk behavior may be accurate, the results have to been seen with caution in light of potential social desirability bias. Third, the study did not include the descriptive norms of the father or mother, although alcohol consumption is a behavior that is influenced by family modelling [66]. We therefore recommend future research to also regard descriptive norms of all relevant socialisation agents. Likewise, the study built on the TPB framework, but it was not the aim of the study to test it as originally proposed. Future studies could extend this line of research by combining theory testing approaches with machine learning approaches. Fourth, although the dataset was split half, and strictly separated model training from model testing, the study cannot prove a causal relationship. A longitudinal or experimental design would be necessary to overcome this limit. Despite these limitations, we do believe that the study adds substantially to the literature.

## 7. Conclusions

Overall, the present study leads to three conclusions. First, there are four concepts that are the most important predictors for sharing drunk references on social media in our sample, namely, the mother’s injunctive norms towards alcohol consumption, peers’ and best friends’ descriptive norms towards alcohol consumption, and willingness to drink alcohol. Second, XG boosting provided the most accurate predictions among the different regression tree algorithms. Third, social media literacy interventions targeting the family system might be an effective method to reduce the sharing of drunk references via social media among adolescents.

## Figures and Tables

**Table 1:** Reliability Indices.

Latent Variable	α	ω_1_	AVE	Low. Loading
Attitude towards Alcohol	0.84	0.84	0.44	0.49
Peers’ injunctive Norms	0.87	0.87	0.54	0.56
Best Friends’ injunctive Norms	0.88	0.90	0.61	0.50
Exposure to Drunk References	0.90	0.92	0.55	0.44
Father’s injunctive Norms	0.81	0.85	0.55	0.36
Mother’s injunctive Norms	0.78	0.84	0.54	0.32
Peers’ descriptive Norms	0.93	0.93	0.67	0.75
Best Friends’ descriptive Norms	0.95	0.95	0.72	0.80
Sensation Seeking	0.74	0.75	0.28	0.25
Sharing Drunk References	0.88	0.91	0.54	0.27
Sensitivity to Peer Pressure	0.79	0.80	0.29	0.39
Social Comparison Orientation	0.80	0.80	0.28	0.29
Fear of negative Evaluation	0.91	0.91	0.48	0.39
Willingness to drink Alcohol	0.91	0.92	0.68	0.31

α refers to Cronbach’s Alpha, ω_1_ to Raykov’s Omega, AVE is the average variance extraction per indicator and the lowest loading was computed using the cfa function in lavaan [48].

**Table 2:** Means, standard deviations, and correlations.

Variable	*M*	*SD*	1	2	3	4	5	6	7	8	9	10	11	12	13	14	15	16	17	18	19	20
1. age	15.14	2.38																				
2. sex	0.59	0.49	−0.01																			
3. religion	0.57	0.49	0.03	0.02																		
4. sharing drunk ref.	0.22	0.43	0.11 *	−0.01	−0.04																	
5. exposure to drunk ref	0.76	0.78	0.14 *	−0.04	−0.02	0.53 *																
6. willingness to drink	0.00	0.98	0.22 *	0.01	−0.02	0.41 *	0.49 *															
7. descriptive norms best friends	0.00	0.98	0.28 *	−0.00	0.00	0.42 *	0.53 *	0.69 *														
8. descriptive norms peers	0.00	0.97	0.30 *	0.14 *	0.03	0.28 *	0.38 *	0.51 *	0.69 *													
9. injunctive norms best friends	0.00	0.95	0.19 *	−0.11 *	−0.05 *	0.33 *	0.47 *	0.63 *	0.74 *	0.52 *												
10. injunctive norms peers	0.00	0.94	0.15 *	−0.02	0.01	0.16 *	0.25 *	0.33 *	0.38 *	0.58 *	0.56 *											
11. injunctive norms mother	0.00	0.92	0.20 *	0.00	−0.08 *	0.36 *	0.39 *	0.53 *	0.52 *	0.40 *	0.55 *	0.35 *										
12. injunctive norms father	0.00	0.93	0.19 *	−0.04	−0.06 *	0.32 *	0.37 *	0.50 *	0.52 *	0.40 *	0.54 *	0.36 *	0.78 *									
13. attitude towards alcohol	0.00	0.92	0.10 *	−0.04	−0.00	0.33 *	0.37 *	0.67 *	0.52 *	0.38 *	0.55 *	0.31 *	0.44 *	0.44 *								
14. sensation seeking	0.00	0.89	0.06 *	−0.06 *	−0.06 *	0.29 *	0.37 *	0.46 *	0.35 *	0.19 *	0.37 *	0.15 *	0.21 *	0.21 *	0.38 *							
15. Facebook use	3.44	2.26	0.23 *	0.07 *	0.07 *	0.26 *	0.36 *	0.42 *	0.46 *	0.43 *	0.36 *	0.27 *	0.36 *	0.33 *	0.34 *	0.15 *						
16. Instagram use	5.19	1.76	0.03	0.15 *	−0.02	0.14 *	0.24 *	0.24 *	0.23 *	0.18 *	0.19 *	0.07 *	0.14 *	0.10 *	0.22 *	0.27 *	0.23 *					
17. Snapchat use	4.99	2.01	0.06 *	0.15 *	0.04	0.20 *	0.30 *	0.34 *	0.32 *	0.25 *	0.26 *	0.10 *	0.19 *	0.16 *	0.29 *	0.27 *	0.29 *	0.48 *				
18. Whatsapp use	3.68	2.12	−0.07 *	0.04	−0.01	−0.06 *	−0.02	−0.21 *	−0.19 *	−0.19 *	−0.19 *	−0.13 *	−0.06 *	−0.06 *	−0.12 *	−0.10 *	−0.11 *	0.04	0.05 *			
19. sensitivity to peer pressure	0.00	0.90	0.02	−0.07 *	0.00	0.26 *	0.24 *	0.31 *	0.22 *	0.13 *	0.27 *	0.15 *	0.14 *	0.17 *	0.33 *	0.34 *	0.16 *	0.09 *	0.13 *	−0.06 *		
20. fear of negative evaluation	0.00	0.96	−0.00	0.31 *	0.01	−0.01	−0.04	0.04	0.03	0.11 *	0.00	0.08 *	−0.04	−0.04	0.04	−0.07 *	0.12 *	0.05	0.05 *	−0.04	0.28 *	
21. social comparison orientation	0.00	0.91	−0.01	0.12 *	0.01	0.04	0.04	0.07 *	0.07 *	0.09 *	0.07 *	0.08 *	−0.01	0.01	0.12 *	0.07 *	0.09 *	0.05 *	0.03	−0.04	0.35 *	0.59 *

Note. M and SD are used to represent mean and standard deviation, respectively. * indicates *p* < 0.05.

**Table 3:** Performance of the Models.

Accuracy Metrics by Approach	Full Dataset	Pruned Dataset
Pruned Tree MSE	0.170	0.170
Pruned Tree R^2^	0.517	0.517
Bagged Tree MSE	0.156	0.159
Bagged Tree R^2^	0.411	0.369
Random Forest MSE	0.154	0.155
Random Forest R^2^	0.444	0.396
Extreme Gradient Boosting MSE	0.076	0.061
Extreme Gradient Boosting R^2^	0.841	0.576

Note. MSE refers to Mean Square Error.

## Data Availability

The datset used for this publication is available under the open science framework (https://osf.io/sda8g/ accessed on 26 September 2021).

## References

[1] Nesi J., Rothenberg W.A., Hussong A.M., Jackson K.M. (2017). Friends’ Alcohol-Related Social Networking Site Activity Predicts Escalations in Adolescent Drinking: Mediation by Peer Norms. J. Adolesc. Health.

[2] Jackson K.M., Janssen T., Cox M.J., Colby S.M., Barnett N.P., Sargent J. (2021). Mechanisms Underlying Associations between Media Alcohol Exposure, Parenting, and Early Adolescent Drinking: A Moderated Sequential Mediation Model. J. Youth Adolesc..

[3] Geusens F., Beullens K. (2018). The Association between Social Networking Sites and Alcohol Abuse among Belgian Adolescents. J. Media Psychol..

[4] Curtis B.L., Lookatch S.J., Ramo D.E., McKay J.R., Feinn R.S., Kranzler H.R. (2018). Meta-Analysis of the Association of Alcohol-Related Social Media Use with Alcohol Consumption and Alcohol-Related Problems in Adolescents and Young Adults. Alcohol. Clin. Exp. Res..

[5] Zeigler D.W., Wang C.C., Yoast R.A., Dickinson B.D., McCaffree M.A., Robinowitz C.B., Sterling M.L. (2005). The neurocognitive effects of alcohol on adolescents and college students. Prev. Med..

[6] White A., Hingson R. (2013). The burden of alcohol use: Excessive alcohol consumption and related consequences among college students. Alcohol Res..

[7] Phan T.-T., Muralidhar S., Gatica-Perez D., Mayora O., Forti S. (2019). #Drink or #Drunk: Multimodal Signals and Drinking Practices on Instagram. Proceedings of the 13th EAI International Conference on Pervasive Computing Technologies for Healthcare—Pervasive Health’19.

[8] Niland P., Lyons A.C., Goodwin I., Hutton F. (2014). ‘See it doesn’t lo ok pretty does it?’ Young adults’ airbrushed drinking practices on Facebook. Psychol. Health.

[9] Atkinson A.M., Sumnall H.R. (2016). ‘If I don’t look good, it just doesn’t go up’: A qualitative study of young women’s drinking cultures and practices on Social Network Sites. Int. J. Drug Policy.

[10] Litt D.M., Lewis M.A., Spiro E.S., Aulck L., Waldron K.A., Head-Corliss M.K., Swanson A. (2018). #drunktwitter: Examining the relations between alcohol-related Twitter content and alcohol willingness and use among underage young adults. Drug Alcohol Depend..

[11] Hendriks H., van den Putte B., Gebhardt W.A. (2018). Alcoholposts on Social Networking Sites: The Alcoholpost-Typology. Cyberpsychol. Behav. Soc. Netw..

[12] Erevik E.K., Torsheim T., Vedaa Ø., Andreassen C.S., Pallesen S. (2017). Sharing of Alcohol-Related Content on Social Networking Sites: Frequency, Content, and Correlates. J. Stud. Alcohol Drugs.

[13] Geusens F., Beullens K. (2017). The reciprocal associations between sharing alcohol references on social networking sites and binge drinking: A longitudinal study among late adolescents. Comput. Hum. Behav..

[14] Ajzen I. (1991). The theory of planned behavior. Organ. Behav. Hum. Decis. Process..

[15] Kern C., Klausch T., Kreuter F. (2019). Tree-based Machine Learning Methods for Survey Research. Surv. Res. Methods.

[16] Rosiers J. (2019). VAD-Leerlingenbevraging: In het Kader van een Drugbeleid op School Syntheserapport Schooljaar 2017–2018. https://www.vad.be/assets/syntheserapport_leerlingenbevraging_2018-2019.

[17] Vandendriessche K., De Marez L. (2019). Digimeter 2019: Digitale Mediatrends in Vlaanderen.

[18] Geber S., Baumann E., Czerwinski F., Klimmt C. (2019). The Effects of Social Norms among Peer Groups on Risk Behavior: A Multilevel Approach to Differentiate Perceived and Collective Norms. Commun. Res..

[19] Borsari B., Carey K.B. (2003). Descriptive and injunctive norms in college drinking: A meta-analytic integration. J. Stud. Alcohol.

[20] Geusens F., Beullens K. (2019). A Longitudinal Examination of the Moderating Influence of Peer and Parental Socialization on Alcohol-Related Social Media Self-Effects Among Late Adolescents. Media Psychol..

[21] Beullens K., Vandenbosch L. (2016). A Conditional Process Analysis on the Relationship between the Use of Social Networking Sites, Attitudes, Peer Norms, and Adolescents’ Intentions to Consume Alcohol. Media Psychol..

[22] Hendriks H., Gebhardt W.A., van den Putte B. (2017). Alcohol-Related Posts from Young People on Social Networking Sites: Content and Motivations. Cyberpsychol. Behav. Soc. Netw..

[23] Gannon-Loew K.E., Eickhoff J.C., Moreno M.A. (2016). The Relationship between Attitude, Social Norms and Alcohol Use: A Longitudinal Analysis Using Facebook. J. Adolesc. Health.

[24] Teunissen H.A., Kuntsche E., Scholte R.H.J., Spijkerman R., Prinstein M.J., Engels R.C.M.E. (2016). Friends’ drinking norms and male adolescents’ alcohol consumption: The moderating role of performance-based peer influence susceptibility. J. Adolesc..

[25] Geusens F., Bigman-Galimore C.A., Beullens K. (2020). Identifying At-Risk Youth. EJHC.

[26] Vogel E.A., Rose J.P., Okdie B.M., Eckles K., Franz B. (2015). Who compares and despairs? The effect of social comparison orientation on social media use and its outcomes. Personal. Individ. Differ..

[27] Reer F., Tang W.Y., Quandt T. (2019). Psychosocial well-being and social media engagement: The mediating roles of social comparison orientation and fear of missing out. New Media Soc..

[28] Khurana A., Bleakley A., Ellithorpe M.E., Hennessy M., Jamieson P.E., Weitz I. (2019). Sensation Seeking and Impulsivity Can Increase Exposure to Risky Media and Moderate Its Effects on Adolescent Risk Behaviors. Prev. Sci..

[29] Davies E.L., Martin J., Foxcroft D.R. (2016). Age differences in alcohol prototype perceptions and willingness to drink in U.K. adolescents. Psychol. Health Med..

[30] Anderson K.G., Garcia T.A., Dash G.F. (2017). Drinking Motives and Willingness to Drink Alcohol in Peer Drinking Contexts. Emerging Adulthood.

[31] Geusens F., Beullens K. (2017). Strategic Self-Presentation or Authentic Communication? Predicting Adolescents’ Alcohol References on Social Media. J. Stud. Alcohol Drugs.

[32] Breiman L., Friedman J., Stone Charles J., Olshen R.A. (1984). Classification and Regression Trees.

[33] Tin Kam H. Random Decision Forests. Proceedings of the 3rd International Conference on Document Analysis and Recognition.

[34] Chen T., Guestrin C., Krishnapuram B., Shah M., Smola A., Aggarwal C., Shen D., Rastogi R. (2016). XGBoost. Proceedings of the 22nd ACM SIGKDD International Conference on Knowledge Discovery and Data Mining—KDD’16.

[35] Gray R.M. (2011). Entropy and Information Theory.

[36] Norman P., Armitage C.J., Quigley C. (2007). The theory of planned behavior and binge drinking: Assessing the impact of binge drinker prototypes. Addict. Behav..

[37] Marks Woolfson L., Maguire L. (2010). Binge drinking in a sample of Scottish undergraduate students. J. Youth Stud..

[38] Elliott M.A., Ainsworth K. (2012). Predicting university undergraduates’ binge-drinking behavior: A comparative test of the one- and two-component theories of planned behavior. Addict. Behav..

[39] Bzdok D., Altman N., Krzywinski M. (2018). Statistics versus machine learning. Nat. Methods.

[40] Van Buuren S., Groothuis-Oudshoorn K. (2011). mice: Multivariate Imputation by Chained Equations in R. J. Stat. Softw..

[41] Cronbach L.J. (1951). Coefficient alpha and the internal structure of tests. Psychometrika.

[42] Raykov T. (2001). Estimation of congeneric scale reliability using covariance structure analysis with nonlinear constraints. Br. J. Math. Stat. Psychol..

[43] Gerrard M., Gibbons F.X., Houlihan A.E., Stock M.L., Pomery E.A. (2008). A dual-process approach to health risk decision making: The prototype willingness model. Dev. Rev..

[44] Santor D.A., Messervey D., Kusumakar V. (2000). Measuring Peer Pressure, Popularity, and Conformity in Adolescent Boys and Girls: Predicting School Performance, Sexual Attitudes, and Substance Abuse. J. Youth Adolesc..

[45] Leary M.R. (1983). A Brief Version of the Fear of Negative Evaluation Scale. Pers. Soc. Psychol. Bull..

[46] Buunk A.P., Gibbons F.X., Guimond S. (2006). Social Comparison Orientation: A New Perspective on Those Who Do and Those Who Don’t Compare with Others. Social Comparison and Social Psychology: Understanding Cognition, Intergroup, Relationship, and Culture.

[47] Hoyle R.H., Stephenson M.T., Palmgreen P., Lorch E.P., Donohew R.L. (2002). Reliability and validity of a brief measure of sensation seeking. Personal. Individ. Differ..

[48] Rosseel Y. (2012). lavaan: An R Package for Structural Equation Modeling. J. Stat. Softw..

[49] Ripley B. (2019). Tree: Classification and Regression Trees. https://CRAN.R-project.org/package=tree.

[50] Therneau T., Athkinson B., Ripley B. (2019). rpart: Recursive Partitioning and Regression Trees. https://CRAN.R-project.org/package=rpart.

[51] Breiman L. (2001). Random Forests. Mach. Learn..

[52] Landry M. (2018). Machine Learning with R and H_2_O, Mountain View. https://www.h2o.ai/wp-content/uploads/2018/01/RBooklet.pdf.

[53] LeDell E., Gill N., Aiello S., Fu A., Candel A., Click C., Kraljevic T., Nykodym T., Aboyoun P., Kurka M. (2020). H_2_O: R Interface for the ‘H_2_O’ Scalable Machine Learning Platform. https://CRAN.R-project.org/package=h2o.

[54] Rhodes N., Ewoldsen D.R., Shen L., Monahan J.L., Eno C. (2014). The Accessibility of Family and Peer Norms in Young Adolescent Risk Behavior. Commun. Res..

[55] Stockdale L.A., Coyne S.M. (2020). Bored and online: Reasons for using social media, problematic social networking site use, and behavioral outcomes across the transition from adolescence to emerging adulthood. J. Adolesc..

[56] Elam K.K., Sternberg A., Waddell J.T., Blake A.J., Chassin L. (2020). Mother and Father Prescription Opioid Misuse, Alcohol Use Disorder, and Parent Knowledge in Pathways to Adolescent Alcohol Use. J Youth Adolesc..

[57] Hastings P.D., McShane K.E., Parker R., Ladha F. (2007). Ready to make nice: Parental socialization of young sons’ and daughters’ prosocial behaviors with peers. J. Genet. Psychol..

[58] Yoon D. (2020). Peer-relationship patterns and their association with types of child abuse and adolescent risk behaviors among youth at-risk of maltreatment. J. Adolesc..

[59] Ridout B., Campbell A., Ellis L. (2012). ‘Off your Face(book)’: Alcohol in online social identity construction and its relation to problem drinking in university students. Drug Alcohol Rev..

[60] Dempsey R.C., McAlaney J., Bewick B.M. (2018). A Critical Appraisal of the Social Norms Approach as an Interventional Strategy for Health-Related Behavior and Attitude Change. Front. Psychol..

[61] Vanassche S., Sodermans A.K., Matthijs K., Swicegood G. (2014). The Effects of Family Type, Family Relationships and Parental Role Models on Delinquency and Alcohol Use among Flemish Adolescents. J. Child Fam. Stud..

[62] Xie X., Gai X., Zhou Y. (2019). A meta-analysis of media literacy interventions for deviant behaviors. Comput. Educ..

[63] Pinkleton B.E., Austin E.W., Hobbs R., Mihailidis P. (2019). Media Literacy and Alcohol Abuse Reduction. The International Encyclopedia of Media Literacy.

[64] Bronfenbrenner U. (1992). Ecological systems theory. Six Theories of Child Development: Revised Formulations and Current Issues.

[65] Flisher A.J., Evans J., Muller M., Lombard C. (2004). Brief report: Test-retest reliability of self-reported adolescent risk behaviour. J. Adolesc..

[66] Pedersen W., von Soest T. (2013). Socialization to binge drinking: A population-based, longitudinal study with emphasis on parental influences. Drug Alcohol Depend..

